# Phosphorylation of the Human DNA Glycosylase NEIL2 Is Affected by Oxidative Stress and Modulates Its Activity

**DOI:** 10.3390/antiox12020355

**Published:** 2023-02-02

**Authors:** Camilla Myrup Holst, Nanna Brøndum Andersen, Vibeke Thinggaard, Mine Tilken, Sofie Lautrup, Cinzia Tesauro, Tinna Stevnsner

**Affiliations:** 1 Department of Molecular Biology & Genetics, Aarhus University, 8000 Aarhus, Denmark; 2 Department of Clinical Molecular Biology, University of Oslo and Akershus University Hospital, 1478 Lørenskog, Norway

**Keywords:** base excision repair, cyclin-dependent kinase 5, post-translational modifications, protein kinase C

## Abstract

The DNA glycosylase NEIL2 plays a central role in maintaining genome integrity, in particular during oxidative stress, by recognizing oxidized base lesions and initiating repair of these via the base excision repair (BER) pathway. Post-translational modifications are important molecular switches that regulate and coordinate the BER pathway, and thereby enable a rapid and fine-tuned response to DNA damage. Here, we report for the first time that human NEIL2 is regulated by phosphorylation. We demonstrate that NEIL2 is phosphorylated by the two kinases cyclin-dependent kinase 5 (CDK5) and protein kinase C (PKC) in vitro and in human SH-SY5Y neuroblastoma cells. The phosphorylation of NEIL2 by PKC causes a substantial reduction in NEIL2 repair activity, while CDK5 does not directly alter the enzymatic activity of NEIL2 in vitro, suggesting distinct modes of regulating NEIL2 function by the two kinases. Interestingly, we show a rapid dephosphorylation of NEIL2 in response to oxidative stress in SH-SY5Y cells. This points to phosphorylation as an important modulator of NEIL2 function in this cellular model, not least during oxidative stress.

## 1. Introduction

The genome is continuously challenged by attacks from reactive oxygen species (ROS) resulting in a variety of oxidative DNA base lesions and strand breaks. To sustain proper cellular function and viability, genomic integrity is maintained by a battery of DNA repair enzymes. The predominant DNA repair pathway involved in resolving ROS-induced lesions is the base excision repair (BER) pathway. In mammals, five bifunctional DNA glycosylases belonging to two structurally distinct families can recognize oxidized base lesions and initiate the BER pathway. All five DNA glycosylases are expressed in the brain [1,2]. They play an important role in responding to the high oxidative DNA damage load encountered within the brain due to its high oxygen consumption and ROS production compared to other tissues (reviewed in [3,4]). Endonuclease III homolog 1 (NTHL1) and 8-oxoguanine-DNA glycosylase (OGG1) belong to the Nth superfamily and preferentially remove oxidized purines and pyrimidines, respectively, in double-stranded DNA. The three DNA glycosylases Nei-like 1, 2, and 3 (NEIL1-3) are members of the Fpg/Nei superfamily, and they exhibit a distinct but overlapping substrate specificity and recognize both purine and pyrimidine oxidation products (reviewed in [5]). Unlike NTHL1 and OGG1, which are only active on duplex DNA, NEIL1-3 can recognize and excise oxidized bases from single-stranded DNA and preferably bubble-structured DNA [6,7]. NEIL2 prefers cytosine-derived lesions such as 5-hydroxyuracil (5-OHU), a common oxidation product, but also has activity towards other oxidized lesions [7,8,9,10]. The DNA glycosylases catalyze cleavage of the N-glycosylic bond between the DNA backbone and the damaged base, whereby the base is excised. Subsequently, the intrinsic apurinic/apyrimidinic (AP) lyase activity, present in the bifunctional DNA glycosylases, catalyzes the hydrolysis of the 3′-phosphodiesterbond at the newly generated AP site, resulting in a single-strand break that is further processed in downstream BER.

NEIL1 expression is upregulated during S-phase and was originally implicated in replication-associated repair, but was later also shown to be involved in transcription-associated DNA repair [11,12,13,14]. In contrast, NEIL2 expression is cell cycle-independent, and NEIL2 preferentially repairs oxidized bases in a transcription-associated manner [15,16]. In the postmitotic brain, proficient repair, of especially transcribed DNA, is vital to preserve sequence fidelity and genomic integrity, whereas the fidelity of the non-transcribed genome in terminally differentiated, non-dividing neurons is less crucial. Due to the inherent high accessibility of transcriptionally active DNA, it is particularly prone to oxidative DNA damage. Accordingly, aged *Neil2*-null mice accumulate oxidized DNA bases in transcribed regions of the genome [15], pointing to NEIL2 as an important contributor to the maintenance of genomic integrity in transcribed DNA.

The first crystal structure of NEIL2 was recently determined in the mammalian species *Monodelphis domestica* (Mdo), while the structure of human NEIL2 still remains to be resolved [17]. Like the remainder of the members of the Fpg/Nei superfamily, MdoNEIL2 consists of an N-terminal domain connected by a domain linker to the C-terminal domain, which contains the conserved helix-two-turns-helix (H2TH) and zinc finger DNA binding motifs. Surprisingly, MdoNEIL2 contains an unusual internal disordered region within the N-terminal domain predicted to be even larger in human NEIL2. Disordered regions serving as an interface for protein interactions and post-translational modifications (PTMs) is a well-known phenomenon for early mammalian BER enzymes; however, they normally occur as terminal extensions (reviewed in [18]). NEIL2 interacts with the downstream BER pathway proteins DNA Polymerase Beta (POLB), DNA Ligase III (LIG3), and X-Ray Repair Cross Complementing 1 (XRCC1) via its N-terminal domain and is associated with these as well as Polynucleotide Kinase 3′-Phosphatase (PNKP) in a multi-component BER complex in mammalian cells [19]. Moreover, the Cockayne Syndrome Complementation Group B (CSB) protein can take part in this complex and interact with and stimulate the activity of NEIL2 [15,20]. NEIL2, in concert with PNKP, also plays a vital role in the maintenance of the mitochondrial genome [21]. Several non-canonical functions of NEIL2 have been reported, including an involvement in DNA demethylation [22,23] and a role as an anti-inflammatory factor during different infections [15,24,25,26].

PTMs are pivotal molecular switches facilitating a rapid spatiotemporal response to, e.g., DNA damage. PTMs provide a means to regulate the protein that is modified in many ways; for example, by altering its binding to interaction partners, modifying its ability to bind DNA, subcellular localization, enzyme activity, and protein stability. Thus, PTMs also play an important role in regulating and coordinating the BER pathway. Phosphorylation followed by acetylation are the most common PTMs reported for BER enzymes (reviewed in [27,28]). Apart from NEIL3, all of the oxidized base-specific DNA glycosylases have been described to be modified by PTMs. NEIL1 has been reported to be acetylated, phosphorylated, and ubiquitylated, thereby regulating its enzymatic activity and binding to chromatin, including association with the actively transcribed regions of the genome and protein stability, respectively [12,29,30,31]. Conversely, the regulation of NEIL2 by PTMs is largely unknown. One study reported NEIL2 to be acetylated by the transcriptional coactivator p300, causing a downregulation in NEIL2 activity [32], while the occurrence of the most common PTM on other BER proteins, phosphorylation, has not yet been investigated. 

Here, we report that NEIL2 is phosphorylated in the human neuroblastoma cell line SH-SY5Y, and that the protein is dephosphorylated in response to acute oxidative stress in this cellular model. We show that protein kinase C (PKC) and the neuron-associated kinase cyclin-dependent kinase 5 (CDK5) phosphorylate NEIL2 in vitro and in SH-SY5Y cells. PKC-mediated phosphorylation decreases NEIL2 activity in vitro, whereas CDK5-mediated phosphorylation does not directly affect the activity of NEIL2 in vitro. This demonstrates that phosphorylation of NEIL2 by different kinases modulates NEIL2 in distinct ways. Our results suggest that phosphorylation plays a role in the regulation of NEIL2 in SH-SY5Y cells, not least in the context of oxidative stress, and point to PKC and CDK5 as two important kinases in this regulatory context.

## 2. Materials and Methods

### 2.1. Cell Culture and Stably Transfected Cell Lines

SH-SY5Y human neuroblastoma cells were obtained from the American Type Culture Collection (Manassas, VA, USA). SH-SY5Y cells were cultured in DMEM/F-12 (1:1) with GlutaMAX supplement (Gibco, #31331028) (Life Technologies, Paisley Park, UK) and supplemented with 10% fetal bovine serum (FBS) and 500 µg/mL penicillin-streptomycin (P/S) at 37 °C in a humidified atmosphere of 5% CO_2_. Cells were subcultured every 2–4 days following harvest by trypsinization. C-terminally His_6_-tagged human NEIL2 expression plasmid (pET22b (+)) was kindly provided by Professor Magnar Bjørås. The open reading frame (ORF) of the C-terminally His_6_-tagged NEIL2 gene was subcloned into the pcDNA3.1.D expression vector utilizing the Directional TOPO cloning kit from Thermo Scientific (Invitrogen, Waltham, MA, USA) according to manufacturer’s protocol. A schematic map of the pcDNA3.1D expression vector can be seen in Figure S1A. Plasmid was extracted with EndoFree Plasmid MaxiKit from Qiagen (Aarhus, DK). For transfection, the cells were grown to around 90% confluency. One hour prior to transfection, the media was replaced with transfection media (DMEM/F-12 (1:1) with GlutaMAX supplement and 5% FBS, without antibiotics). Ninety percentage confluent cells in a 100 mm (diameter) Petri dish were transfected with 24 µg NEIL2 expression vector in the presence of 60 µL lipofectamine reagent from Invitrogen (Waltham, MA, USA)) according to manufacturer’s protocol. Sixteen hours post-transfection, cells were washed once in PBS and media replaced with fresh transfection media. Twenty-four hours post-transfection, media was replaced with selection media (DMEM/F-12 1:1 with GlutaMAX supplement, 10% FBS, 500 µg/mL P/S, 600 µg/mL geneticin (Gibco, #10131-035) (Life Technologies, Paisley Park, UK), 40% conditioned medium). Geneticin-resistant cells were selected over 5 weeks with regular replacement of media every 3–4 days and expanded when reaching high confluency. The optimal concentration of geneticin for selection of stably transfected SH-SY5Y was determined beforehand by generation of a kill curve with non-transfected SH-SY5Y cells. The lowest concentration of geneticin at which all cells were dead in 10 days was chosen. Monoclonal stably transfected cells were isolated from the population of polyclonal stably transfected cells by limited dilution. Cells were seeded in 100 µL selection media in 96-well plates at a concentration of 5 cells/mL and cultured for about 8 weeks with regular replacement of selection media and expansion when reaching high confluency. After selection of stably transfected cells, the geneticin concentration was lowered to 300 µg/mL for maintenance of cells. Genomic integration of the pcDNA3.1.D expression vector in the stably transfected cells as well as the presence of intact sequence from the promoter behind NEIL2 to the polyA signal was confirmed by PCR, as seen in Figure S1B. His_6_-tagged NEIL2 protein levels were analyzed by Western blotting. The monoclonal cell line expressing the highest level of His_6_-tagged NEIL2 was utilized for downstream analyses, as seen in Figure S1C.

### 2.2. Metabolic Labeling with [^32^P]Orthophosphate

Monoclonal His_6_-tagged NEIL2-expressing SH-SY5Y cells were seeded in a 100 mm Petri dish and grown to around 80–90% confluency in DMEM/F-12 (1:1) supplemented with GlutaMAX, 10% FBS, 500 µg/mL P/S, and 300 µg/mL geneticin. Cells were starved for 1 h in phosphate-free DMEM (Gibco, #11971025) (Life Technologies, Paisley Park, UK) and supplemented with 2.5% FBS to exhaust the pool of endogenous phosphate, followed by 3 h incubation in 3 mL phosphate-free DMEM containing 2.5% FBS and 0.1 mCi/mL media of carrier-free [^32^P]orthophosphate (PerkinElmer, #NEX053H) (Waltham, MA, USA). To induce oxidative stress, 1 mL phosphate-free DMEM −/+ H_2_O_2_ was added to the labeling reaction to a final concentration of 0 or 500 µM H_2_O_2_ (as indicated) during the last 30 min of the incubation. For analysis with kinase inhibitors, cells were pre-treated with 10 µM Gö 6983 (Abcam, #144414) (Cambridge, UK), 10 µM roscovitine (Sigma, #R7772) (Merck, Darmstadt, Germany), or vehicle (0.1% DMSO) for 1 h prior to starvation in DMEM/F12 media. Ten µM inhibitor was also included during starvation and labeling. After the treatment, cells were washed three times in ice-cold 1xTBS (10 mM Tris-HCl pH 7.5, 150 mM NaCl), collected by scraping, and cell pellets frozen at −70 °C until further processing. Cells were lysed in 200 µL IP lysis buffer (25 mM Tris-HCl pH 7.4, 150 mM NaCl, 1 mM EDTA, 1% NP-40, 5% glycerol, 1% protease inhibitor cocktail (Merck, #539134) (Darmstadt, Germany), 2% phosphatase inhibitor cocktail 2 (Sigma, #P5726) (Merck, Darmstadt, Germany), and 2% phosphatase inhibitor cocktail 3 (Sigma, #P0044) (Merck, Darmstadt, Germany)) for 10 min at 4 °C. Cell debris was removed by centrifugation at 13,000× *g* for 10 min at 4 °C. Meanwhile, 50 µL (1.5 mg) per IP of Dynabeads™ Protein A (#10001D, Invitrogen) (Waltham, MA, USA) was incubated with anti-His-tag antibody (Cell Signaling, #12698, 1:100) (Danvers, MA, USA) in 200 µL PBST for 10 min at room temperature, followed by a single wash in PBST. Antibody-bound beads were incubated with cell lysate for 1 h at 4 °C and, subsequently, beads were washed three times in ice-cold IP lysis buffer. Proteins were eluted from the beads in 1xSDS loading dye (1X NuPage LDS loading buffer from Life Technologies (Paisely Park, UK) with 100 mM DTT) by incubation at 70 °C for 10 min. The immunoprecipitated proteins were incubated at 90 °C for 10 min and separated in a 7% Tris-acetate (TA) gel with 1xTA running buffer (Life Technologies) for 1 h 15 min at 150 V. The gel was fixated for 15 min in 10% acetic acid and 50% ethanol. Phosphorylated proteins were visualized by phosphoimaging (BioRad, Hercules, CA, USA). Total protein was visualized using the SilverQuest™ silver staining kit from Invitrogen (Waltham, MA, USA) according to the manufacturer’s protocol.

### 2.3. Immunoblotting Analysis

Proteins were separated by SDS-PAGE and transferred to a PVDF membrane by dry transfer using the iBlot dry blotting system from Invitrogen (Waltham, MA, USA). Membranes were blocked for 1 h at room temperature in 5% *w*/*v* milk or BSA in TBS-T (20 mM Tris-HCl pH 8.0, 137 mM NaCl, 0.05% Tween), followed by incubation with primary antibody overnight at 4 °C. Primary antibodies: anti-His-tag antibody (#A00186, GenScript, 1:5000) (Piscataway, NJ, USA), anti-His-tag antibody (#12698, Cell Signaling, 1:1000) (Danvers, MA, USA), anti-NEIL2 antibody (#124106, Abcam, 1:500) (Cambridge, UK), anti-P35 antibody (anti-p35, SantaCruz, #sc-518009, 1:2500) (Dallas, TX, USA), and anti-actin antibody (#A2228, Sigma, 1:10,000) (Merck, Darmstadt, Germany). Subsequently, the membrane was washed in TBS-T and incubated with secondary anti-mouse IgG Horseradish Peroxidase Linked (GE Healthcare, #NA931, 1:5000) (Chicago, IL, USA) or anti-rabbit IgG Horseradish Peroxidase Linked (GE Healthcare, #NA934, 1:5000) (Chicago, IL, USA) antibody for 1 h at room temperature, followed by additional washing in TBS-T and detection with ECL Prime Western Blotting Detection Reagent from GE Healthcare (Chicago, IL, USA) according to the manufacturer’s protocol.

### 2.4. Bioinformatics

In silico predictions of phosphorylation sites and kinases were conducted with the NetPhos database (v. 3.1) [33,34] with the human NEIL2 protein sequence (Q969S2-1). The PhosphoSitePlus database (v. 6.5.9.3) [35] was used to search for known phosphorylation sites in the literature.

### 2.5. Cloning and Purification of NEIL2

A C-terminally His_6_-tagged human NEIL2 expression plasmid (pET22b (+)) was used for the expression and purification of NEIL2. NEIL2 was purified as described in [20] with some modifications; BL21-CodonPlus (DE3)-RIL *E. coli* strain from Agilent Technologies (Glostrup, DK) was transformed with the NEIL2 expression plasmid. The NEIL2 and tRNA expression plasmids were maintained by 100 µg/mL ampicillin and 50 µg/mL chloramphenicol, respectively. See Figure S2 for a representative SDS-PAGE from NEIL2 purification.

### 2.6. In Vitro Kinase Assays

Two hundred ng recombinant NEIL2 was incubated with either 100 ng CDK5 (#C0745, Sigma) (Merck, Darmstadt, Germany) or 25 ng PKC (PKC purified from rat brain, #V526A, Promega (Fitchburg, WI, USA), recombinant PKCγ (#K4518, Sigma), or recombinant PKCα (P1782, Sigma) (Merck, Darmstadt, Germany) (as indicated)) in a 25 µL reaction volume containing either 1xCDK5 buffer (5 mM MOPS pH 7.2, 2.5 mM glycerol-2-phosphate, 5 mM MgCl_2_, 1 mM EGTA, 1 mM EDTA, and 50 µM DTT) or 1xPKC buffer (20 mM HEPES pH 7.4, 10 mM MgCl_2_, and 100 µM CaCl_2_), respectively, for 40 min at 30 °C. The reaction also included 20 nM unlabeled ATP and 25 µCi/mL [^32^P]-ATP (Hartmann Analytic, #HP-601-10) (Braunschweig, Germany). For subsequent NEIL2 activity assays, phosphorylation reactions were instead conducted with 80 µM ATP and incubated for 10 min at 30 °C, followed by immediate continuation to the activity assay. Reactions were stopped by addition of SDS loading buffer (1X NuPage LDS loading buffer from Life Technologies (Paisley Park, UK) with 100 mM DTT) and incubated at 95 °C for 10 min. Proteins were separated by SDS-PAGE in 7% TA gels with 1xTA running buffer from Life Technologies (Paisley Park, UK) for 1 h at 150 V. Gels were fixated for 15 min in 10% acetic acid and 50% ethanol and phosphorylated proteins visualized by phosphoimaging (BioRad) (Hercules, CA, USA). Total protein was visualized using the SilverQuest™ silver staining kit from Invitrogen (Waltham, MA, USA) according to the manufacturer’s protocol. For the immunoprecipitation of NEIL2 after CDK5/P25-mediated phosphorylation, samples were boiled for 10 min at 95 °C to eliminate protein interactions. 1.5 mg of Dynabeads™ Protein G (#10003D) from Invitrogen (Waltham, MA, USA) was incubated with anti-His-tag antibody (Genscript, #A00186, 1:100) (Piscataway, NJ, USA) in 200 µL PBST for 10 min at room temperature, followed by a single wash in PBST. Antibody-bound beads were incubated with samples from the kinase assay for 1 h at room temperature. Subsequently, beads were washed three times in PBST. Proteins were eluted from the beads in 1xSDS loading dye (1X NuPage LDS loading buffer from Life Technologies (Paisley Park, UK) with 100 mM DTT) by incubation at 70 °C for 10 min. The immunoprecipitated proteins were incubated at 90 °C for 10 min and run in a 7% TA gel with 1xTA running buffer from Life Technologies (Paisley Park, UK) for 1 h 15 min at 150 V. The gel was fixated for 15 min in 10% acetic acid and 50% ethanol. Phosphorylated proteins were visualized by phosphoimaging (BioRad) (Hercules, CA, USA). Total protein was visualized using the SilverQuest™ silver staining kit from Invitrogen (Waltham, MA, USA) according to the manufacturer’s protocol. Western blotting was also conducted with the immunoprecipitated samples as described in the immunoblotting section. 

### 2.7. Radiolabeling and Annealing of Oligonucleotides

Oligonucleotides were purchased from Sigma (Merck, Darmstadt, Germany) (Table S1) and 5′-end labeled by incubation with [^32^P]-ATP (Hartmann Analytic, #HP-601-10) (Braunschweig, Germany) and T4 polynucleotide kinase from Thermo Scientific (Paisley Park, UK) for 90 min at 37 °C in 1xforward buffer A from Thermo Scientific (Paisley Park, UK). Reactions were stopped by incubation at 90 °C for 1 min. Free [^32^P]-ATP was removed by the use of G50 microspin columns from GE Healthcare (Chicago, IL, USA) according to manufacturer’s protocol. Labeled oligonucleotide was annealed to a partially complementary oligonucleotide in 175 mM KCl and 100 mM EDTA with 5 min incubation at 90 °C, followed by gradual cooling overnight.

### 2.8. In Vitro DNA Repair Activity Assays

An amount of 0.5–2.5 nM recombinant NEIL2 was incubated with 2 nM oligonucleotide substrate (control or lesion-containing, see Table S1) for 15 min at 37 °C in a buffer containing 40 mM Tris-HCl pH 8.0, 4 mM MgCl_2_, 30 mM NaCl, 5 mM DTT, 0.5 mM β-mercaptoethanol, 100 µg/mL BSA, 100 µM EDTA, and 5% glycerol. Reactions were stopped by addition of 2xformaldehyde loading buffer (80% formaldehyde with 100 mM NaOH to cleave residual AP sites) and boiled for 5 min at 95 °C. Non-cleaved substrate and the shorter repair product were separated on a 20% denaturing polyacrylamide gel and visualized by phosphoimaging (BioRad). Percentage incision was calculated as the amount of product relative to the sum of substrate and product. For the determination of kinetic constants, 2.5 nM NEIL2 was incubated with increasing concentrations of substrate (0.4–12.8 nM). Reactions were incubated for 5 min (V0). Kinetic constants were determined with GraphPad Prism (v. 9.4.1) software using the Michaelis–Menten model (Y = [NEIL2] ∗ k_cat_ ∗ X/(K_M_ + X)). The substrate concentration was plotted against the velocity (nM product/min), and non-linear regression was performed to determine K_M_ and k_cat_. For experiments with CSB stimulation of NEIL2, CSB was purified as previously described [36] and included in the repair reaction in a molar ratio of 1:2 for NEIL2:CSB.

### 2.9. Proximity Ligation Assay

Cells were seeded on poly-d-lysine/laminin B coated cover glass and grown for 48 h in DMEM/F-12 (1:1) supplemented with GlutaMAX, 10% FBS, and 500 µg/mL P/S. For stably transfected cells, 300 µg/mL geneticin was added. After 48 h, the media was changed to differentiation media (Neurobasal media (#21103-49, Gibco) (Paisley Park, UK)) supplemented with 0.5 mM GlutaMAX (#35050-038, Gibco) (Paisley Park, UK), 1xB27 (#1704044, Gibco) (Paisley Park, UK), and 1% P/S (300 µg/mL geneticin for stabile transfectants). Ten µM retinoic acid (#R2625, Sigma) (Merck, Darmstadt, Germany) was added to the differentiation media for the first 5 days, followed by 5 days with 10 ng/mL BDNF (#450-02, PreproTech) (Thermo Scientific, Paisley Park, UK). Cells were fixed for 15 min in 4% formaldehyde and permeabilized in 0.25% Triton-X for 10 min. The proximity ligation assay (PLA) was conducted with the Duolink In Situ Red Starter Kit Mouse/Rabbit (#DUO92101, Sigma) (Merck, Darmstadt, Germany) according to manufacturer’s protocol. Cells were incubated with primary antibodies as indicated: anti-His-tag (Cell Signaling, #12698, 1:600) (Danvers, MA, USA), pan anti-PKC (Santa Cruz, #sc-17769, 1:1000) (Dallas, TX, USA), and anti-CDK5 (Santa Cruz, #sc-6247, 1:100) (Dallas, TX, USA). PLA signals were visualized using an Axio Observer microscope and Zen Pro software (Zeiss, Oberkochen, Germany).

### 2.10. Statistics

Statistical analysis was performed using GraphPad Prism software (v. 9.4.1). Statistical comparisons between two groups were performed with Student’s *t*-test (equal variances) and with one-way ANOVA (Dunnett’s post hoc test) for multiple comparisons. *p*-values < 0.05 were considered statistically significant. *: *p* ≤ 0.05; **: *p* ≤ 0.01; ***: *p* ≤ 0.001, ****: *p* ≤ 0.0001.

## 3. Results

### 3.1. NEIL2 Is Phosphorylated in SH-SY5Y Cells

**Figure 1:**
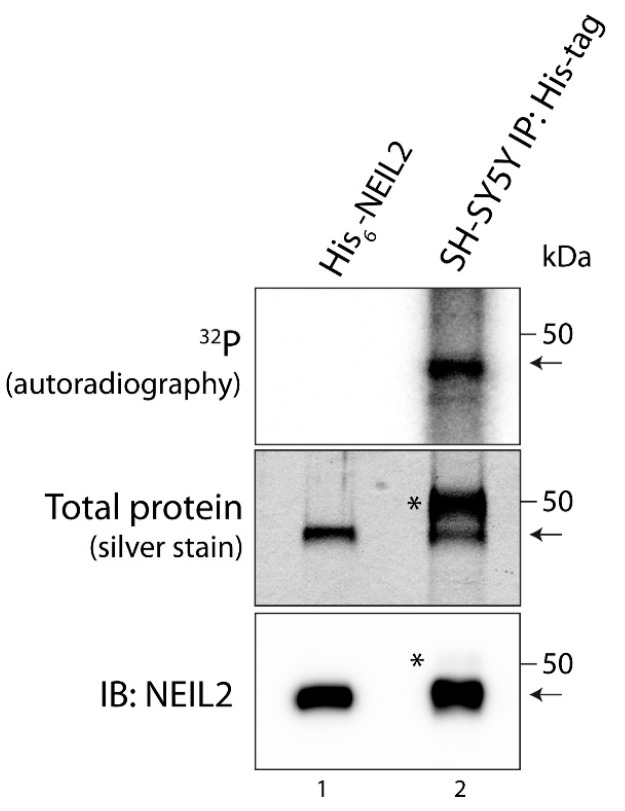
NEIL2 is phosphorylated in SH-SY5Y cells. SH-SY5Y cells expressing His-tagged NEIL2 were incubated with [^32^P]orthophosphate for 3 h. Subsequently, cells were lysed and His-tagged NEIL2 was immunoprecipitated with anti-His-tag antibody (Cell Signaling, #12698). Proteins were separated by SDS-PAGE and phosphorylation detected by autoradiography. Proteins in the SDS-PAGE were stained by silver staining. The band close to 50 kDa was confirmed to be NEIL2 by immunoblotting (IB) using anti-NEIL2 antibody (Abcam, #124106). Bands marked with * represent rabbit IgG heavy chain from immunoprecipitation. Arrows point to NEIL2. Lane 1: Purified His_6_-tagged NEIL2 loaded in SDS-PAGE and immunoblotting as a control. Lane 2: Immunoprecipitated NEIL2 from SH-SY5Y cells.

In order to investigate whether NEIL2 is regulated by phosphorylation in cells, we expressed His-tagged NEIL2 in the human neuroblastoma SH-SY5Y cell line. SH-SY5Y cells are commonly used as a neuronal cell model [37,38] and have previously been used for studying NEIL2 function [21,39]. We transfected SH-SY5Y cells with the mammalian expression vector pcDNA3.1 expressing C-terminally His-tagged NEIL2 and generated monoclonal stabile transfectants by selection with geneticin (Figure S1). To examine phosphorylation, we performed metabolic labeling in the His-tagged NEIL2 expressing SH-SY5Y cells with radioactively labeled inorganic phosphate. The cells were incubated with [^32^P]orthophosphate for 3 h. Subsequently, proteins were isolated from the cells and NEIL2 was immunoprecipitated by the use of an anti-His-tag antibody and subjected to SDS-PAGE. The precipitated protein exhibited a size comparable to purified, recombinant NEIL2, included as a control in the SDS-PAGE (Figure 1, middle panel). Moreover, it was confirmed that the precipitated protein is NEIL2 by immunoblotting with an anti-NEIL2 antibody (Figure 1, lower panel). We did not observe notable unspecific pulldown in the immunoprecipitation, suggesting that NEIL2 accounts for the vast majority of protein present at the band corresponding to the size of NEIL2, although minor contributions from proteins with a similar molecular weight cannot be ruled out. Autoradiography analysis clearly demonstrated that NEIL2 is indeed phosphorylated in SH-SY5Y cells by the appearance of an intense band corresponding to the size of NEIL2 in the SDS-PAGE (Figure 1, upper panel).

### 3.2. In Silico Prediction of Responsible Kinases

Based on the observed phosphorylation of NEIL2 in SH-SY5Y cells, we investigated which kinases NEIL2 has the potential to serve as a substrate for. In order to predict kinases, an in silico analysis based on the primary amino acid sequence of NEIL2 was performed in the NetPhos database [33,34]. Several potential NEIL2-targeting kinases were identified and the top three kinases, based on highest prediction scores and number of predicted sites, were PKC, protein kinase A (PKA), and CDK5 (Table 1).

NEIL2 contained eight potential phosphosites for PKC and five potential sites for PKA and CDK5, respectively. Based on the in silico analysis, we investigated whether the highest-ranking kinase, PKC, as well as the neuron-associated kinase CDK5 are involved in phosphorylation of NEIL2. These two kinases are predicted to phosphorylate distinct sites in NEIL2 and both kinases are expressed and active in SH-SY5Y cells [40,41,42].

### 3.3. NEIL2 Is Phosphorylated by PKC and Phosphorylation Decreases the Repair Activity of NEIL2 In Vitro

**Figure 2:**
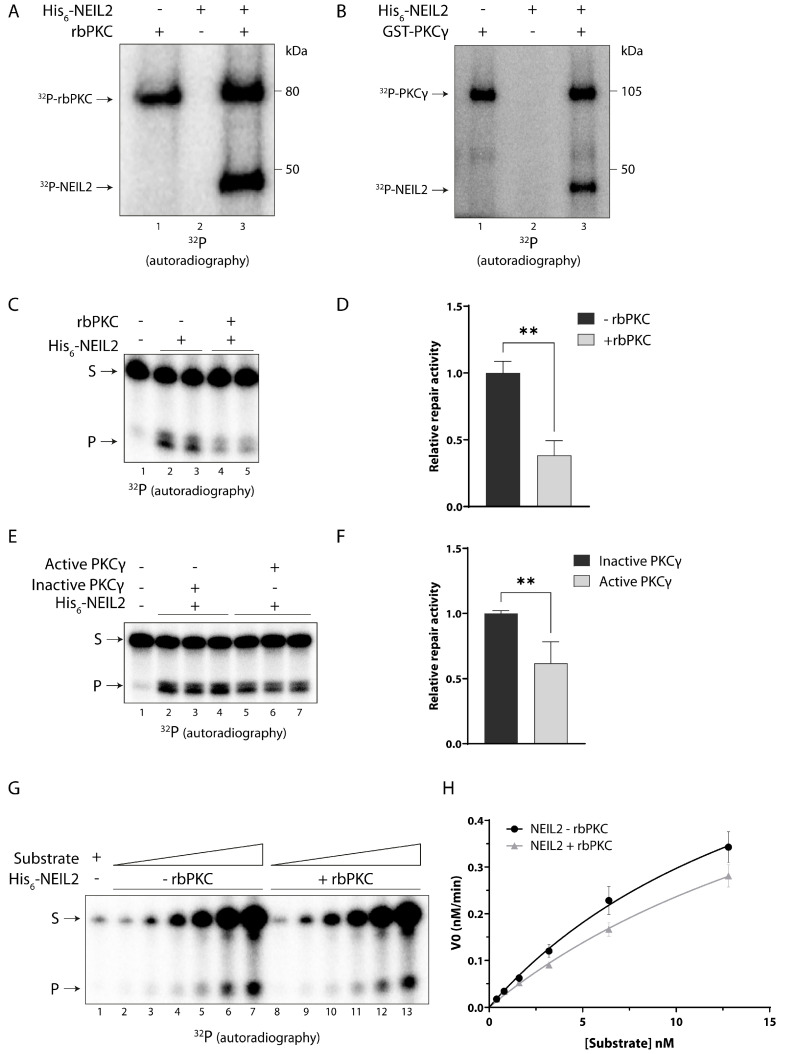
PKC-mediated phosphorylation of NEIL2 reduces its activity in vitro. (**A**,**B**) Purified NEIL2 was incubated with kinase, as indicated, in the presence of [^32^P]-ATP for 45 min at 30 °C and proteins were separated by SDS-PAGE for autoradiography. (**A**) 200 ng of purified recombinant NEIL2 was incubated with 25 ng PKC purified from rat brain (rbPKC). PKC underwent autophosphorylation and can be seen as a band around 80 kDa. Phosphorylation of NEIL2 can be seen as a band close to 50 kDa. (**B**) 200 ng of purified recombinant NEIL2 was incubated with 25 ng recombinant GST-tagged PKCγ. Autophosphorylation of PKCγ can be seen as a band around 105 kDa. NEIL2 was incubated with PKC, rbPKC (**C**,**D**,**G**,**H**) or recombinant PKCγ (**E**,**F**), respectively, in the presence of unlabeled ATP for 10 min at 30 °C. Subsequently, non-phosphorylated (incubated for 10 min at 30 °C without kinase or with heat inactivated kinase) or phosphorylated NEIL2 was incubated with a 5′-end ^32^P-labeled oligonucleotide substrate containing a 5-OHU lesion in an 11nt bubble (5-OHU B11) for 15 min. Substrate and repair product were separated in a denaturing polyacrylamide gel and visualized by autoradiography. (**G**,**H**) For kinetic analysis, NEIL2 was incubated with an increasing concentration of the 5-OHU B11 substrate for 5 min (V0). S: substrate, P: product. Data are displayed as mean + SD and represent at least three independent experiments. **: *p* ≤ 0.01.

PKC is a serine/threonine (Ser/Thr) kinase with ten different isoforms, which display differences in structural features, cofactors required for activation, and expression patterns (reviewed in [43]). PKC is predicted to phosphorylate eight sites at both Ser and Thr residues in human NEIL2 (Table 1). These sites are positioned across the sequence of the NEIL2 protein in both the N-terminal and C-terminal domain, and some of them occur in predicted structural motifs of NEIL2 including the disordered region, domain linker, and the zinc finger motif (Figure S3). In general, phosphosites tend to be conserved between mammalian species [44,45]. Accordingly, the majority of the predicted PKC phosphosites were conserved between human, mouse, and rat, and many of the sites are also fully conserved to *M. domestica* and the more distantly related non-mammalian vertebrate *X. tropicalis* (Figure S4). The latter has also been used for studying NEIL1 and NEIL2 function [22,46]. In order to investigate whether NEIL2 is a substrate for PKC, the kinase was incubated with recombinant human His_6_-tagged NEIL2 in the presence of [^32^P]-ATP in an in vitro kinase assay. For the assay, we initially used PKC purified from rat brain (rbPKC). The rbPKC primarily consists of the α, β, and γ isoforms of PKC with lesser amounts of the isoforms δ and ζ. After incubation with rbPKC, a radioactive band was detected close to 50 kDa corresponding to the size of NEIL2 (Figure 2A, lane 3), thereby demonstrating that PKC phosphorylates NEIL2 in vitro. In addition, we also demonstrated that both recombinant GST-tagged PKCγ and PKCα, respectively, can phosphorylate NEIL2 in vitro (Figure 2B, lane 3 and Figure S5A). PKCβ and other isoforms were not examined in this study.

Based on the observations that PKC phosphorylates NEIL2 in vitro, we investigated whether the phosphorylation affects the activity of NEIL2 by an in vitro DNA repair activity assay. In this assay, purified recombinant human NEIL2 was incubated with a double-stranded oligonucleotide containing a 5-OHU lesion in an internal 11 bp bubble structure (5-OHU B11 substrate) (Figure S6A). NEIL2 activity results in a nick at the site of the DNA lesion and the repair product can be separated from the full-length substrate in a denaturing gel and visualized by phosphoimaging due to a radiolabel on the same strand as the lesion (Figure S6B). Phosphorylation by rbPKC caused a significant reduction in NEIL2 activity by more than 50% (Figure 2C,D). A reduction in NEIL2 activity was also observed after phosphorylation by PKCγ (Figure 2E,F). On the other hand, PKCα-mediated phosphorylation of NEIL2 did not significantly affect the activity of NEIL2 (Figure S5B), despite a similar degree of phosphorylation by the two isoforms (Figure S5A). This suggests that phosphorylation by different PKC isoforms can have differential effects on NEIL2 activity and points to PKCγ, a neuron-specific PKC isoform, as one of the major PKC isoforms in the rbPKC involved in altering the activity of NEIL2.

A kinetic analysis was conducted to investigate the mechanism behind PKC-mediated reduction in NEIL2 activity. The analysis demonstrated that the decrease in NEIL2 activity by rbPKC-mediated phosphorylation is associated with an increased K_M_ value of NEIL2, whereas k_cat_ remains unchanged by phosphorylation (Figure 2G–H and Table 2). This suggests that the decrease in NEIL2 activity by phosphorylation in vitro is caused by a reduction in the affinity of NEIL2 towards the 5-OHU B11 substrate. Notably, the kinetic parameters are similar to those previously reported for unphosphorylated NEIL2 when incubated with the same substrate [7].

### 3.4. CSB Stimulates NEIL2 Activity Similarly in the Presence and Absence of PKC Phosphorylation In Vitro

**Figure 3:**
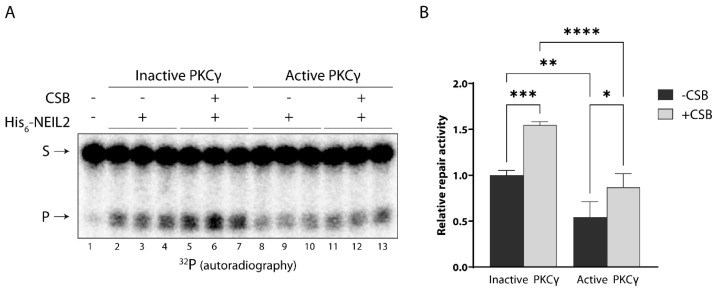
CSB stimulates NEIL2 repair activity similarly with and without PKC-mediated phosphorylation of NEIL2 in vitro. (**A**) NEIL2 was incubated with active or heat inactivated PKCγ, respectively, in the presence of unlabeled ATP for 10 min at 30 °C. Phosphorylated or non-phosphorylated NEIL2 was incubated with 5′-end ^32^P-labeled 5-OHU B11 substrate for 15 min in the presence or absence of CSB (molar ratio of 1:2 for NEIL2:CSB). Substrate and repair product were separated in a denaturing polyacrylamide gel and visualized by autoradiography. S: substrate, P: product. (**B**) Quantification of relative NEIL2 repair activity. Data are displayed as mean + SD and represent three independent experiments. *: *p* ≤ 0.05; **: *p* ≤ 0.01; ***: *p* ≤ 0.001, ****: *p* ≤ 0.0001.

NEIL2 can occur in multicomponent BER repair complexes in mammalian cells together with the DNA repair associated protein CSB. We have previously demonstrated that CSB interacts with and stimulates the activity of NEIL2 [20]. In light of the observed PKC-mediated reduction in NEIL2 activity and as phosphorylation often modulates protein–protein interactions, we wondered whether PKC-mediated phosphorylation of NEIL2 would affect the ability of CSB to stimulate NEIL2 repair activity. To investigate this, NEIL2 was phosphorylated by PKC and subsequently incubated with CSB and the 5-OHU B11 substrate. PKC phosphorylation of NEIL2 significantly reduced its activity both in the absence and presence of CSB. Furthermore, for both the unphosphorylated and phosphorylated form of NEIL2, its activity was significantly increased 1.5-fold in the presence of CSB (Figure 3). This suggests that CSB interacts with and stimulates NEIL2 to a similar extent whether NEIL2 has been subjected to PKC phosphorylation or not. However, as the NEIL2 activity resulting from combined PKC and CSB treatment is significantly lower than NEIL2 only treated with CSB, these results also indicate that CSB cannot compensate for the reduction in NEIL2 activity caused by PKC-mediated phosphorylation under the conditions examined here, possibly due to an effect of the phosphorylation.

### 3.5. NEIL2 Is Phosphorylated by CDK5 In Vitro, but It Does Not Affect NEIL2 Activity

CDK5 is a proline (Pro)-directed Ser/Thr kinase with the minimal consensus sequence [Ser*/Thr*-Pro] (* represents the phosphosite). CDK5 is an atypical member of the Cdk family as it is not directly involved in regulating the cell cycle. Its regulatory subunit P35 (P25 after calpain cleavage) is essential for CDK5 activity and is mainly expressed in the central nervous system (reviewed in [47]). CDK5 is predicted to phosphorylate five sites in NEIL2 (Table 1). Four of the sites are positioned in the N-terminal domain, including two in the disordered region and one positioned in the interdomain linker (Figure S3). One of the sites, S68, along with the minimal CDK5 consensus criterion, is conserved, while the other predicted phosphosites are not (Figure S4). Interestingly, two of the sites, S68 and T70, have previously been reported in several high throughput screens of the human phosphoproteome [48,49,50,51,52], indicating that they may be phosphorylated, although further investigation has not been conducted.

**Figure 4:**
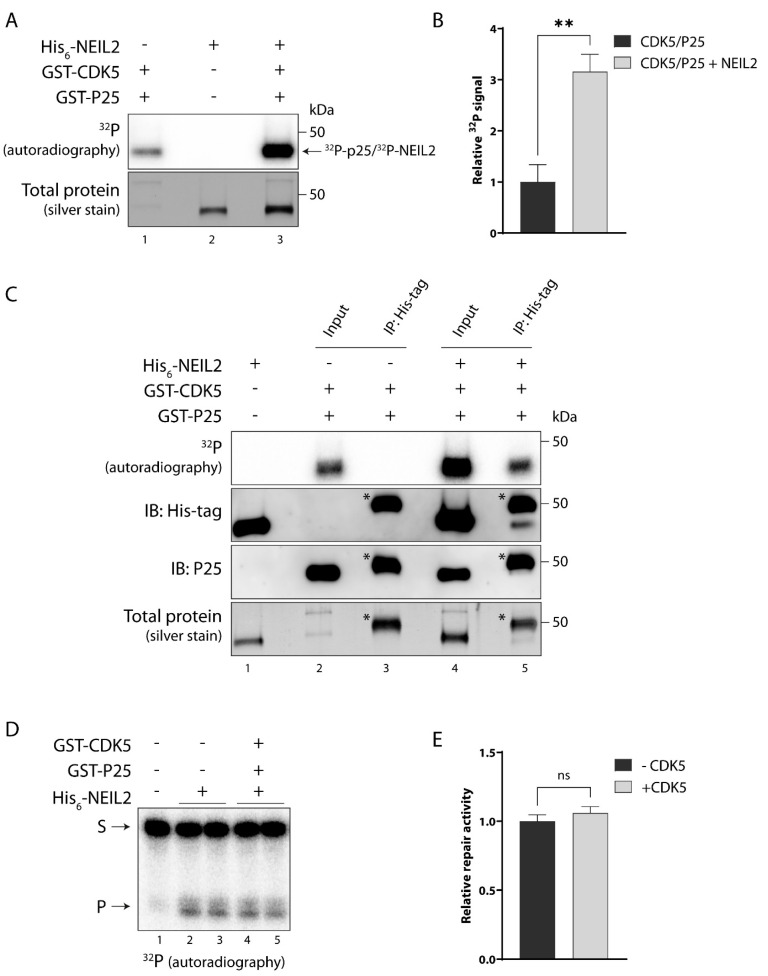
NEIL2 is phosphorylated by CDK5 without affecting NEIL2 activity in vitro. (**A**–**C**) Purified NEIL2 was incubated with kinase, as indicated, in the presence of [^32^P]-ATP for 45 min at 30 °C, and proteins were separated by SDS-PAGE for autoradiography. The phosphorylation signal was analyzed by autoradiography and total protein was stained by silver staining. (**A**) 200 ng of purified NEIL2 was incubated with 100 ng recombinant GST-tagged CDK5/P25. CDK5 phosphorylates its regulatory subunit P25 which can be seen as a band just below 50 kDa due to the GST-tag on P25. The ^32^P signal at 50 kDa is therefore the sum of NEIL2 and P25 phosphorylation. Total protein was stained by silver staining. (**B**) Quantification of ^32^P signal at 50 kDa for CDK5/P25 by itself and CDK5/P25 incubated with NEIL2. Data are displayed as mean + SD and represent three independent experiments. (**C**) To discriminate the ^32^P signal from NEIL2 and P25, NEIL2 was immunoprecipitated after incubation with CDK5/P25. 200 ng of purified NEIL2 was incubated with 100 ng recombinant GST-tagged CDK5/P25 in the presence of [^32^P]-ATP for 45 min at 30 °C, followed by incubation at 95 °C for 10 min to eliminate protein interactions. NEIL2 was immunoprecipitated with anti-His-tag antibody (GenScript, #A00186) (lane 4 + 5). Immunoprecipitation in a sample containing only CDK5/P25 (lane 2 + 3) was included as a negative control. Successful isolation of NEIL2 from P25 by immunoprecipitation was checked by immunoblotting (IB) using anti-His-tag antibody (GenScript, #A00186) and anti-P25 antibody (SantaCruz, #sc-518009). Bands marked with * represent mouse IgG heavy chain. (**D**,**E**) NEIL2 was incubated with CDK5/P25 in the presence of unlabeled ATP for 10 min at 30 °C. Subsequently, non-phosphorylated (incubated for 10 min at 30 °C without kinase) or phosphorylated NEIL2 was incubated with a 5′-end ^32^P-labeled oligonucleotide substrate containing a 5-OHU lesion in an 11nt bubble (5OHU B11) for 15 min. Substrate and repair product were separated in a denaturing polyacrylamide gel and visualized by autoradiography. S: substrate, P: product. (**E**) Quantification of relative NEIL2 repair activity. Data are displayed as mean + SD and represent five independent experiments. **: *p* ≤ 0.01.

To examine whether NEIL2 is a substrate for CDK5, we incubated NEIL2 with recombinant GST-tagged CDK5 together with the regulatory subunit P25 and in the presence of [^32^P]-ATP. Due to the GST-tag on P25, P25 migrates at a similar size as NEIL2 during SDS-PAGE; furthermore, because CDK5 phosphorylates P25, a radioactive band close to 50 kDa was detected even in the absence of NEIL2 (Figure 4A, lane 1). However, the addition of NEIL2 to CDK5/P25 caused a significant 3-fold increase in the phosphorylation signal close to the 50 kDa position (Figure 4A, lane 3 and Figure 4B), indicating that NEIL2 is phosphorylated by CDK5. We confirmed the CDK5-mediated phosphorylation of NEIL2 by immunoprecipitating NEIL2 with anti-His-tag antibody following the in vitro kinase assay, and thereby eliminated the signal from P25 (Figure 4C). The immunoblots demonstrate that His-tagged NEIL2 is present in the precipitate, although a large amount of NEIL2 was lost during the immunoprecipitation, whereas P25 was removed (Figure 4C, two middle panels, lane 5). After the immunoprecipitation of NEIL2, a radioactive band was still present close to 50 kDa in the absence of P25 (Figure 4C, lane 5, upper panel), whereas no radioactive band was observed in a control immunoprecipitation with a sample containing CDK5/P25 without NEIL2 (Figure 4C, lane 3). This confirms that CDK5 phosphorylates NEIL2 in vitro. Based on the observation that CDK5 phosphorylates NEIL2 in vitro, we investigated whether phosphorylation affects the activity of NEIL2 by the same in vitro DNA repair activity assay as described above for PKC. Unlike PKC, CDK5-mediated phosphorylation of NEIL2 did not significantly alter the repair activity of NEIL2 in vitro (Figure 4D,E), demonstrating that CDK5 does not exert a regulatory effect on NEIL2 by directly altering its activity in vitro.

### 3.6. NEIL2 Interacts with and Is Phosphorylated by PKC and CDK5 in SH-SY5Y Cells 

**Figure 5:**
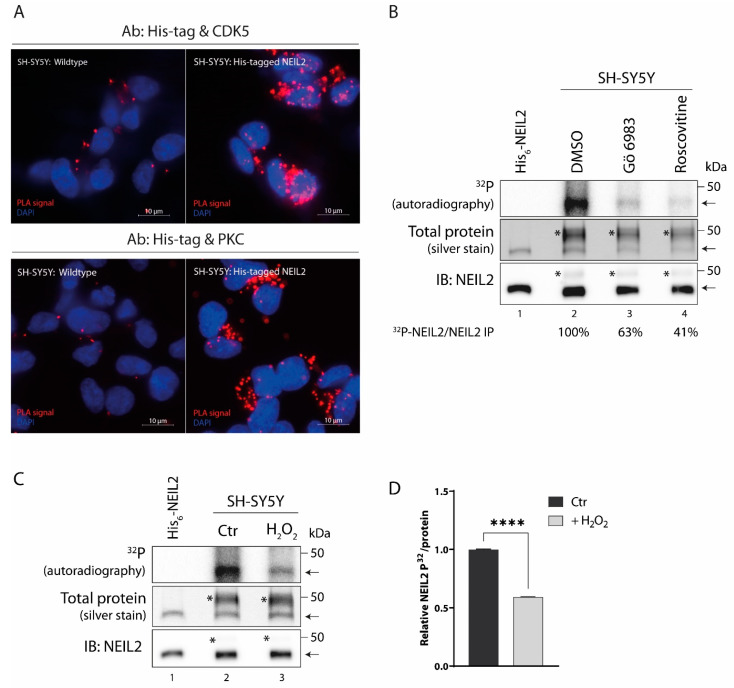
CDK5- and PKC-mediated phosphorylation of NEIL2 and oxidative stress induced dephosphorylation in SH-SY5Y cells. (**A**) In situ PLA detection of NEIL2 interaction with CDK5 and PKC, respectively in SH-SY5Y cells expressing His-tagged NEIL2. Upper panel: PLA with anti-His-tag (rabbit) and anti-CDK5 (mouse). Lower panel: PLA with anti-His-tag (rabbit) and anti-PKC (mouse). Left side: SH-SY5Y wildtype cells. Right side: His-tagged NEIL2-expressing SH-SY5Y cells. (**B**) SH-SY5Y cells expressing His-tagged NEIL2 were pre-treated with vehicle (DMSO), 10 µM Gö 6983 (PKC inhibitor), or 10 µM roscovitine (CDK5 inhibitor) for 2 h and incubated with [^32^P]orthophosphate for 3 h in the presence of vehicle, Gö 6983, or roscovitine, respectively. Cells were lysed and His-tagged NEIL2 was immunoprecipitated with anti-His-tag antibody (Cell Signaling, #12698). Proteins were separated by SDS-PAGE and phosphorylation detected by autoradiography. Proteins in the SDS-PAGE were stained by silver staining. The band close to 50 kDa was confirmed to be NEIL2 by immunoblotting (IB) using anti-NEIL2 antibody (Abcam, #124106). Numbers below figure correspond to ratio of ^32^P signal/immunoprecipitated NEIL2 expressed as percentage relative to DMSO control. Lane 1: Purified His_6_-tagged NEIL2 loaded in SDS-PAGE and immunoblotting as a control. Bands marked with * represent rabbit IgG heavy chain. Arrows point to NEIL2. (**C**) SH-SY5Y cells expressing His-tagged NEIL2 were incubated with [^32^P]orthophosphate for 3 h and during the last 30 min either mock treated or treated with 500 µM H_2_O_2_, as indicated. NEIL2 was immunoprecipitated with anti-His-tag antibody (Cell Signaling, #12698) and analyzed by autoradiography, immunoblotting, and silver staining, as described in (**B**). Bands marked with * represent rabbit IgG heavy chain. Arrows point to NEIL2. (**D**) Quantification of relative ^32^P signal for NEIL2 normalized to the amount of NEIL2 protein measured by silver stain. Data are displayed as mean + SD and represent three independent experiments. ****: *p* ≤ 0.0001.

Based on the in vitro studies, we examined whether the link between NEIL2 phosphorylation and the two kinases, PKC and CDK5, persists in SH-SY5Y cells. To this end, we conducted an in situ proximity ligation assay (PLA). The high sensitivity of this assay provides a mean to observe transient interactions, such as the interaction between a kinase and its target, and at the same time provides information on the presence of the interaction within the cell. To detect NEIL2 in the PLA assay, an antibody against His-tags was utilized. However, anti-His-tag antibodies can cross-react with endogenous nuclear proteins harboring naturally occurring repetitive sequences of His residues in SH-SY5Y cells and other cell lines [53]. The 60 kDa transcription factor YY1 contains an internal stretch of 11 His residues that is recognized by His-tag antibodies [54] and likely contributes to a large part of the cross-reactivity in immunofluorescent stainings. To circumvent this problem, we utilized a rabbit anti-His-tag antibody (Cell Signaling) that only recognizes His-tags fused to the N- or C-terminal of proteins. Immunoprecipitation with this antibody followed by SDS-PAGE and immunoblot analysis showed that NEIL2 is precipitated without the appearance of a 60 kDa band in SH-SY5Y cells, whereas immunoprecipitation with a mouse anti-His-tag antibody (GenScript) that recognizes both external and internal His-tags causes the majority of the precipitate to consist of a 60 kDa protein. For this reason, we only used the rabbit anti-His-tag antibody for the PLA assay. To confirm the specificity of the His-tag antibody, we compared immunofluorescent staining between wildtype and His-tagged NEIL2-expressing SH-SY5Y cells. We did not detect any signal in the wildtype cells, while the His-tagged NEIL2-expressing cells displayed staining with the largest fraction present in the nuclei (Figure S7). This is in accordance with previous reports of the subcellular localization of NEIL2, which localizes both to the nucleus and the mitochondria [8,21]. Wildtype or His-tagged NEIL2-expressing SH-SY5Y cells were differentiated and fixed, permeabilized, and probed with antibodies against His-tag and PKC (pan antibody recognizing all PKC isoforms) or CDK5, respectively. PLA signals were observed both for antibodies against PKC and CDK5, respectively, in combination with the anti-His-tag antibody in the SH-SY5Y NEIL2-expressing cells (Figure 5A, right side of panels). The PLA signals appeared to occur both within and outside the nucleus, suggesting interactions in both the nuclear and mitochondrial compartments. A minor part of the PLA signal was due to unspecific background, as can be seen in the wildtype cells (Figure 5A, left side of panels). This suggests that both of the kinases, PKC and CDK5, interact with His-tagged NEIL2, and since PLA signals are only generated when proteins are in close proximity (≤40 nm) of each other, this indicates a direct protein–protein interaction and not merely co-localization of the proteins.

Based on the observed interaction in SH-SY5Y cells, we next investigated whether the kinases contribute to the phosphorylation of NEIL2 in this cellular model, as observed in Figure 1. To identify responsible kinases, metabolic labeling with radioactively labeled inorganic phosphate was conducted in the presence of vehicle or kinase inhibitors. PKC was inhibited by the addition of 10 µM Gö 6983, a broad-spectrum pan-PKC inhibitor, and CDK5 was inhibited by the addition of 10 µM roscovitine. Due to a high similarity between CDK5 and other CDKs, no specific CDK5 inhibitor currently exists. Roscovitine is the most commonly used compound to inhibit CDK5; however, roscovitine can also inhibit other CDKs, in particular CDK1 and CDK2 [55,56]. The addition of Gö 6983 and roscovitine, respectively, caused a considerable reduction in the phosphorylation signal for NEIL2 (Figure 5B), indicating that kinases inhibited by these inhibitors are responsible for the majority of the phosphorylation of NEIL2. Collectively, the protein interaction and the blockage of phosphorylation by specific kinase inhibitors suggest that CDK5 and PKC play a major role in regulating NEIL2 by phosphorylation in SH-SY5Y cells.

### 3.7. Oxidative Stress Induces Dephosphorylation of NEIL2 in SH-SY5Y Cells 

NEIL2 plays a key role in the repair of oxidized base lesions, which become particularly abundant during oxidative stress due to the imbalance in ROS levels, and many DNA repair enzymes respond to genetic insults through the modulation of their PTMs. We therefore wondered whether the phosphorylation status of NEIL2 would be altered by oxidative stress. To examine the effect of oxidative stress on phosphorylation, we performed metabolic labeling in the His-tagged NEIL2-expressing SH-SY5Y cells. The cells were incubated with [^32^P]orthophosphate for 3 h and during the last 30 min either mock treated or treated with 500 µM H_2_O_2_ to induce oxidative stress. This concentration was chosen as it is within the range previously shown to cause elevated intracellular ROS levels, increased oxidative DNA damage, and induction of the DNA damage response in SH-SY5Y cells with limited impact on cell viability [57,58,59]. As can be seen in Figure 5C, similar amounts of NEIL2 were immunoprecipitated across treatments (Figure 5C, middle and lower panel, lane 2 + 3), and treating cells for 30 min with H_2_O_2_ caused a statistically significant decrease in the level of NEIL2 phosphorylation by 40% (Figure 5C,D). These results demonstrate that NEIL2 is regulated by phosphorylation in SH-SY5Y cells and that NEIL2 is dephosphorylated as a response to acute oxidative stress.

## 4. Discussion

The genome constantly faces endogenous and exogenous sources of DNA damage with an estimated rate of DNA damage in the order of 10^4^–10^5^ DNA lesions per cell per day under normal conditions (reviewed in [60]), and imbalanced ROS levels occurring during oxidative stress greatly increases this burden. The cellular DNA damage response relies heavily on PTMs such as phosphorylations to orchestrate a rapid and temporal response to counteract genotoxic insults and thereby maintain the integrity of the genome. 

Here, we report for the first time that the oxidized base-specific DNA glycosylase, NEIL2, is modulated by phosphorylation in vitro and in SH-SY5Y cells and responds rapidly to oxidative stress by dephosphorylation in this cellular model. Furthermore, it is revealed that two kinases, PKC and CDK5, phosphorylate NEIL2 in vitro and in SH-SY5Y cells. These two kinases appear to be responsible for the majority of NEIL2 phosphorylation in SH-SY5Y cells, albeit significant contribution from other kinases, including other CDKs inhibited by roscovitine, cannot be ruled out. However, these cell cycle-regulating kinases are likely to only play a minimal role in non-dividing neurons. Phosphorylation by PKC caused a substantial reduction in NEIL2 activity in vitro and was associated with a decreased substrate affinity, whereas CDK5-mediated phosphorylation did not directly affect NEIL2 activity in vitro. This illustrates that different NEIL2 phosphorylation events can have differential outcomes. Similar observations have been made for other BER enzymes. Downregulated, upregulated, and unaffected enzymatic activity upon phosphorylation seems to occur equally frequently in the BER pathway (reviewed in [27,61]). For example, CDK4-mediated phosphorylation of the DNA glycosylase OGG1 increases its activity, whereas phosphorylation by PKC or c-Abl does not alter its activity [62]. Likewise, phosphorylation by CDK5 and CKII inhibits the activity of the AP endonuclease APE1, whereas PKC- and CKI-mediated phosphorylation has no effect on its activity [63,64]. Although we did not observe any effect of CDK5-mediated phosphorylation in vitro, it cannot be excluded that phosphorylation by CDK5 affects the activity of NEIL2, directly or indirectly, in vivo. Furthermore, PKC- and CDK5-mediated phosphorylation may also have other biological consequences such as regulation of the association with interaction partners, substrate preferences, spatial distribution within the cells, or protein stability. For instance, although PKC-mediated phosphorylation of OGG1 does not affect its activity, it appears to be involved in OGG1 chromatin-association [65]. Moreover, the phosphorylation of NEIL2 may play a role in one or more of its noncanonical functions, including DNA demethylation and inflammation. Given the involvement of NEIL2 in transcription-associated DNA repair, it could also be speculated whether phosphorylation plays a role in this context, such as by modulating the association with known interaction partners functioning as components of the transcription machinery, including RNAPOLII, TFIIH, and hnRNP-U [15,16]. Of note, the acetylation of NEIL1 strongly regulates NEIL1 occupancy at actively transcribed DNA [12]. Most of the predicted CDK5 and PKC phosphosites are positioned in the N-terminal domain of NEIL2, and several sites in this domain are within the predicted disordered region, known to serve as a hub for protein interactions in other BER enzymes (reviewed in [18]). Interestingly, YB-1, POLB, LIG3, and XRCC1 all interact with NEIL2 via its N-terminal domain [19,66], whereas mapping of the interacting domain for CSB or other transcription-associated interaction partners has not yet been conducted. 

We show that hydrogen peroxide-induced oxidative stress stimulates a rapid dephosphorylation of NEIL2 in SH-SY5Y cells. Oxidative stress has previously been reported to alter NEIL2 function. Accordingly, NEIL2-initated repair of 5-OHU lesions appears to be arranged in a multicomponent repair complex comprised of NEIL2 and the downstream BER proteins PNKP, POLB, LIG3, and XRCC1 [19]. The repair of 5-OHU lesions by this complex is upregulated during oxidative stress in a manner strongly dependent on the non-canonical BER factor, YB-1, which translocates to the nucleus during oxidative stress, partakes in the repair complex, and stimulates NEIL2 activity [66]. Several of the components of the NEIL2 repair complex have also been reported to be regulated by phosphorylation during oxidative stress. Opposite to NEIL2, PNKP is rapidly phosphorylated in response to hydrogen peroxide by the DNA damage-activated kinase ATM, and PNKP phosphorylation is associated with increased stability in PNKP and cellular resistance to oxidative stress [67]. Meanwhile, LIG3 is phosphorylated by CDK2, and oxidative stress induces LIG3 dephosphorylation during S-phase in a way which appears to involve the inhibition of CDK2 in an ATM-dependent manner [68]. This highlights how a similar stimulus can give rise to opposing outcomes regarding PTMs on distinct targets that act in the same pathway. Hyper-phosphorylation in response to DNA damage is a common phenomenon in the DNA damage response [69]. In contrast, besides dephosphorylation of LIG3 during S-phase, NEIL2 dephosphorylation is, to our knowledge, the first report of a general hypo-phosphorylation of a BER enzyme upon oxidative stress. Meanwhile, hypo-phosphorylation in response to hydrogen peroxide-induced oxidative stress has been reported for components of other cellular processes extremely dependent on genomic integrity, including cell cycle progression as well as transcription, as a mechanism to deal with impaired integrity [70,71].

The steady-state level of protein phosphorylation depends on the combined efforts of kinases and phosphatases. The rapid dephosphorylation of NEIL2 upon oxidative stress suggests that a phosphatase is involved, although the repression of CDK5 and/or PKC phosphorylation could also be an alternative or additional mechanism. However, oxidative stress is a major activator of CDK5, and hyperactive CDK5, in turn, can exacerbate the degree of oxidative stress by phosphorylating and inactivating components of the antioxidant defense system [47,72,73]. Additionally, the activation of PKC has also been reported in connection with oxidative stress (reviewed in [74]). However, CDK5 can activate the Ser/Thr phosphatase protein phosphatase 1 (PP1) by phosphorylation of its negative regulator inhibitor-2 (I-2), resulting in I-2 dissociation from PP1 [75]. Accordingly, acute oxidative stress has been reported to concurrently induce increased CDK5 activity and the activation of PP1 by dissociation of phosphorylated I-2 in SH-SY5Y cells and rat hippocampal neurons. This CDK5-activated PP1 facilitates hypo-phosphorylation of the Alzheimer’s disease associated protein, tau, an otherwise known target of CDK5 phosphorylation [76]. It is, thus, tempting to speculate whether a similar paradoxical relationship exists between CDK5 and the phosphorylation-dephosphorylation of NEIL2.

**Figure 6:**
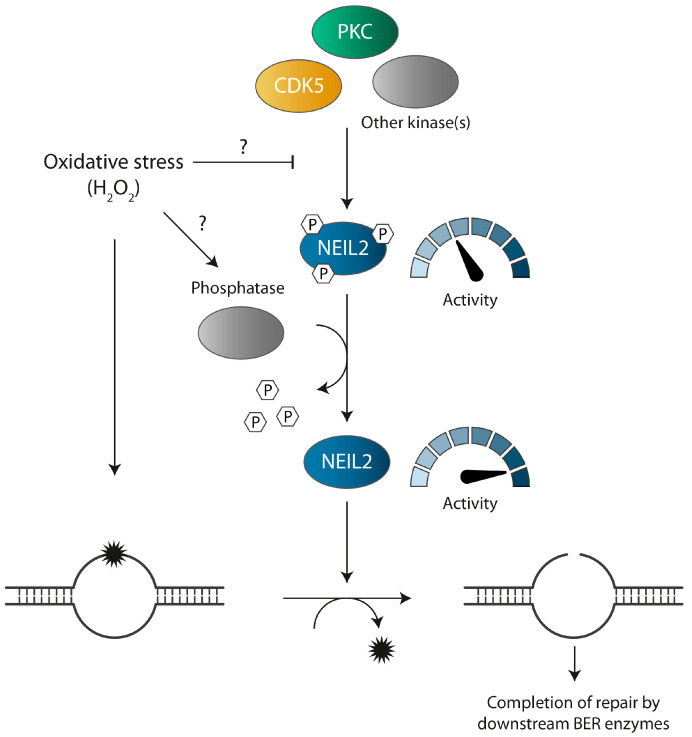
Proposed model for regulation of NEIL2 phosphorylation during oxidative stress. Under normal conditions, NEIL2 is present in a phosphorylated state mediated by protein kinase C (PKC), cyclin-dependent kinase 5 (CDK5), and possibly additional kinases. NEIL2 activity is kept at basal level by PKC phosphorylation. In response to H_2_O_2_-induced oxidative stress, the level of oxidative DNA damage is increased and NEIL2 is dephosphorylated. Dephosphorylation may occur by an increased phosphatase activity and/or inhibition of phosphorylation (indicated by a question mark). Dephosphorylation releases the repressive effect on NEIL2 activity and NEIL2 can efficiently initiate repair of oxidized base lesion via BER.

Based on our findings, we propose a working model in which NEIL2 is kept in a phosphorylated state by PKC, CDK5, and possibly other kinases during normal conditions within the cell, and functions at a basal level able to respond to the regular amount of DNA damage continuously encountered. Upon oxidative stress, the level of oxidized base lesions is elevated in the genome and NEIL2 is dephosphorylated by a mechanism involving a phosphatase and/or inhibition of kinase phosphorylation. This possibly lifts the repressive effect of phosphorylation on NEIL2 activity and facilitates an adequate NEIL2-initiated repair response to counteract the raised threat against the genome (Figure 6). However, the kinetics of NEIL2 dephosphorylation and whether NEIL2 is re-phosphorylated to its default state succeeding oxidative stress remains to be determined. Altogether, our results contribute to the understanding of how reversible PTMs regulate the BER pathway, albeit many questions have yet to be answered to understand how PTMs collectively control and coordinate the BER pathway.

## Figures and Tables

**Table 1:** In silico prediction of NEIL2 phosphorylation sites and responsible kinases.

Kinase	Score Range	No. of Predicted Sites	Predicted Sites
PKC	0.56–0.87	8	S28, S29, T25, S198, S101, T312, S250, S134
PKA	0.51–0.69	5	S134, S39, S162, S36, S29
CDK5	0.50–0.61	5	T191, S68, T70, S162, S187

Predictions were conducted in the NetPhos database. Number of sites are defined by a prediction score >0.5 (recommended cut-off). The top three kinases based on number of predicted sites and scores are displayed. Predicted sites are listed in descending order according to score for each kinase. PKC: protein kinase C. PKA: protein kinase A. CDK5: Cyclin-dependent kinase 5.

**Table 2:** Phosphorylation by rbPKC increases K_M_ of NEIL2.

Kinase	−rbPKC	+rbPKC
K_M_ (nM)	17.98 ± 4.1	25.05 ± 5.6
k_cat_ (×10^3^ nM/min)	33.27 ± 5.1	33.16 ± 5.4
k_cat_/K_M_ (min^−1^ nM^−1^)	1.85 ± 1.2	1.32 ± 1.0

Data are displayed as mean ± SD and represent three independent experiments. rbPKC: rat brain protein kinase C.

## Data Availability

Data are contained within the article and Supplementary Materials.

## Supplementary Materials

The following supporting information can be downloaded at: https://www.mdpi.com/article/10.3390/antiox12020355/s1, Figure S1: Stable integration of pcDNA3.1. NEIL2 expression vector in SH-SY5Y cells; Figure S2: Purification of human C-terminally His_6_-tagged NEIL2; Figure S3: Schematic overview of the position of predicted PKC and CDK5 phosphosites in the human NEIL2 protein; Figure S4: Evolutionary conservation of predicted phosphosites in NEIL2; Figure S5: PKC alpha phosphorylates NEIL2 without altering NEIL2 repair activity in vitro; Figure S6: Principle of the NEIL2 DNA repair assay; Figure S7: Specificity of His-tag antibody in immunofluorescence imaging; Table S1: List of oligonucleotides used for NEIL2 activity assay. Supplementary materials and methods.
