# Supplementary material for: Phosphorylation of the Human DNA Glycosylase NEIL2 Is Affected by Oxidative Stress and Modulates Its Activity
Source: Antioxidants (Basel). 2023 Feb 2;12(2):355. doi: 10.3390/antiox12020355 (PMC9952225; doi:10.3390/antiox12020355)
Supplement: Supplementary file 1 [file antioxidants-12-00355-s001.zip › Figure S1-S7.pdf]

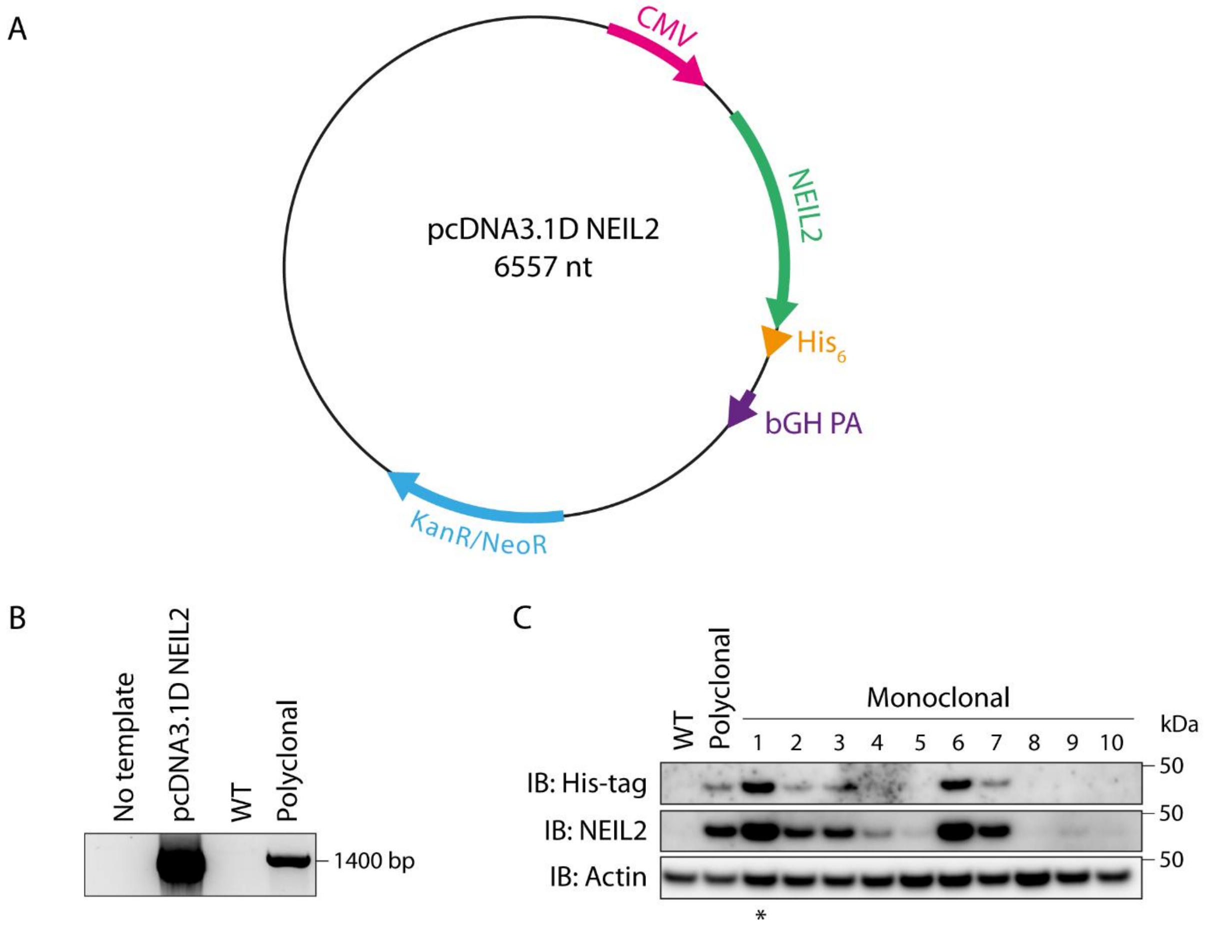

Figure S1 Stable integration of pcDNA3.1 NEIL2 expression vector in SH-SY5Y cells A) Schematic map of the pDNA3.1.D expression vector showing the most relevant vector elements. The vector was used for expression of C-terminally His_6-tagged NEIL2 in SH-SY5Y cells. CMV: cytomegalovirus promoter; bGH PA: bovine growth hormone polyadenylation signal; KanR/NeoR: Kanamycin/Neomycin Resistant Gene (confers geneticin resistance in eukaryotes) B) PCR was conducted with genomic DNA purified from wildtype (WT) and polyclonal stably transfected SH-SY5Y cells and primers targeting the CMV promoter and bGH PA signal, respectively, in the expression vector. Genomic integration of the expression vector will result in a PCR product of 1394 bp. C) Expression level of His_6-tagged NEIL2 protein in WT, polyclonal and monoclonal SH-SY5Y cells was examined by immunoblotting using anti-His-tag antibody (Cell Signaling, #12698), anti-NEIL2 antibody (Abcam, #124106) and anti-Actin antibody (Sigma, #A2228). *: monoclonal cell line utilized for downstream analysis.

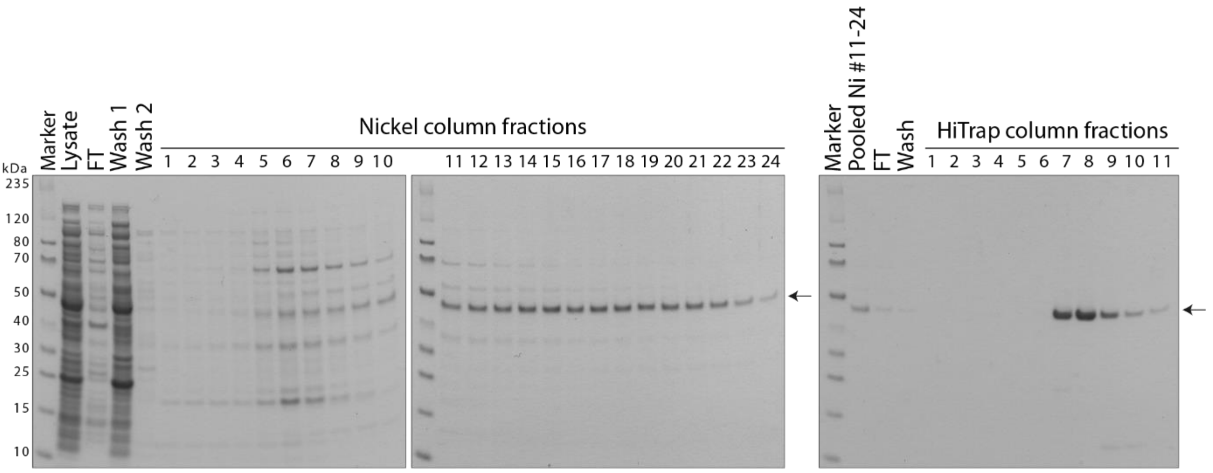

Figure S2 Purification of human C-terminally His$_6$-tagged NEIL2 His$_6$-tagged NEIL2 was expressed from the pET-22b expression vector in *E. coli* and purified on a nickel column. Fractions containing the highest concentration of NEIL2 were pooled and concentrated on a HiTrap column. Fractions from both columns were analysed by SDS-PAGE with Coomassie protein stain. Black arrows indicate the position of NEIL2 in the SDS-PAGE. FT: flow through.

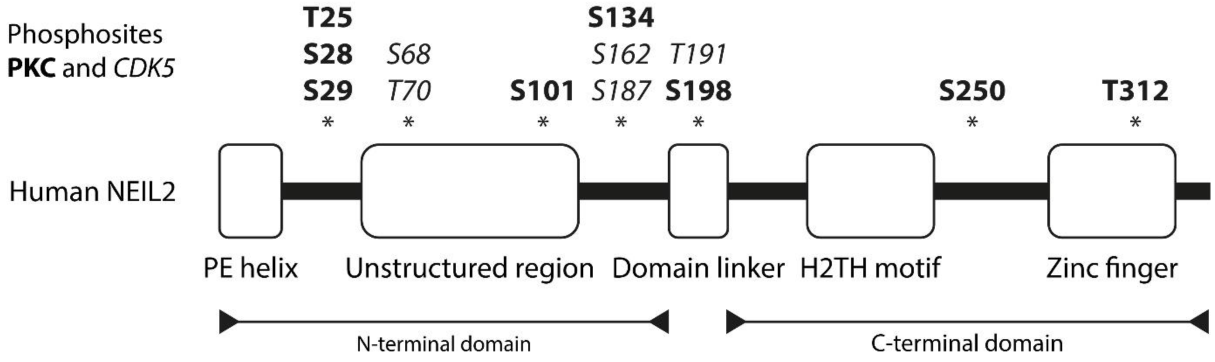

Figure S3 Schematic overview of the position of predicted PKC and CDK5 phosphosites in the human NEIL2 protein Predicted structural features of human NEIL2 are based on comparison with members of the same structural family as the crystal structure of human NEIL2 has yet to be resolved. H2TH: helix 2-tern helix turn. *: approximate position of phosphosites. Bold: predicted PKC phosphosites. Italics: predicted CDK5 phosphosites.

```
Xenopus          MPEGPTVRRFCSLVSPFVGQNVVKVGGSTKKIALQDLQGKQFHYCQVHGKNLYLEFTTKA   60
Monodelphis      MPEGPSLRKFHQLVAPFVGQLVVTVGGNSKKINPNMLEMLRLQDSQVHGKNLYLNFGLTE   60
Human            MPEGPLVRKFHHLVSPFVGQQVVKTGGSSKKLQPASLQSLWLQDTQVHGKKLFLRFDLDE   60
Mouse            MPEGPSVRKFHHLVSPFVGQKVVKTGGSSKKLHPAAFQSLWLQDAQVHGKKLFLRFDPDE   60
Rat              MPEGPSVRKFHHLVSPFVGQKVVKTGGSSKKLHPATFQSLWLQDAQVHGKKLFLQFDPDE   60
                 ***** :*:*  **:***** **..**.:**:     ::    ::  *****:*:*.*

Xenopus          VGNILSGTEDAK--------------EHIDQAG---------GSQKDYSIIQPTSEEHSE   97
Monodelphis      DLGLPESFLLPKHLQKKVRLPKEKSDHKLETTSRLDGQEV-PGSSLAIKALELGEEEKET   119
Human            EMGPPGSSPTPEPPQKEVQKEGAADPKQV---GEPSGQKTLDGSSRSAELVPQGEDDSE-   116
Mouse            EMEPLNSSPQPIQG--MWQ-KEAVDRELA---LGPSAQEPSAGPSGSGEPVPSRSAETY-   113
Rat              EMEPLSRSPQPVQG--MWQ-KEAGNPEPA---LGPSAQETSVGPCGSGEPGPSTSDGPY-   113
                                           .               *     .     .

Xenopus          SLQDADYSEIHMSRWLRFHFGLYGSVRSNEFARAKQANKRGDWRDPTPRLILNFESGGFL   157
Monodelphis      VMPWWLNTSQNSGLWLCFHFGLFGSVRASELSRATKANKRGDWKDPIPRLVLHFAK-GFL   178
Human            -YLERDAPAGDAGRWLRVSFGLFGSVWVNDFSRAKKANKRGDWRDPSPRLVLHFGGGGFL   175
Mouse            -NL-GKIPSADAQRWLEVRFGLFGSIWVNDFSRAKKANKKGDWRDPVPRLVLHFSGGGFL   171
Rat              -DLRGKKPLAEAQRWLEVRFGLFGSIWVNDFSRAKKANKRGDWIDPVPRLVLHFSGGGFL   172
                           .   ** . ***:**:  .:::**.:***:*** ** ***:*:*   ***

Xenopus          VFYNCRMAWCSSPASKPNCDVLSPEFDTEQAVRALSASQPVCFTLMDQKCFSGVGNIIKN   217
Monodelphis      AFYNCRIYWCLGPTVKPTSDILSEEFDRRQALEALKQASPVSYTLLDQRYFAGLGNIIKN   238
Human            AFYNCQLSWSSSPVVTPTCDILSEKFHRGQALEALGQAQPVCYTLLDQRYFSGLGNIIKN   235
Mouse            VFYNCQMSWSPPPVIEPTCDILSEKFHRGQALEALSQAQPVCYTLLDQRYFSGLGNIIKN   231
Rat              AFYNCQMSWSPPPVIEPTCDILSEKFHRGQALEALSQAQPVCYTLLDQRYFSGLGNIIKN   232
                 .****:: *.  *.  *..*:** :*.  **:.**  :.**.:**:**: *:*:******

Xenopus          EILFLEQVHPLCLGSLLPIEKLHSLVSHTLHFTSDWLSSKMKKEALHYHIYMKEYCDKGH   277
Monodelphis      EVLYLARIHPLSLGSCLTPLNLESLLDHVVSFSVGWLQKKLEGKPLHHLIYQKEQCPAGH   298
Human            EALYRAGIHPLSLGSVLSASRREVLVDHVVEFSTAWLQGKFQGRPQHTQVYQKEQCPAGH   295
Mouse            EALYRARIHPLSLGSCLSSSSREALVDHVVEFSKDWLRDKFQGKERHTQIYQKEQCPSGH   291
Rat              EALYRARIHPLSLGSFLSPSSLEALVDHVVEFSTDWLRDKFQGKERHTQIYQKEQCPSGH   292
                 * *:   :***.*** *      . *:.*.: *:  **  *:: .  *  :* ** *  **

Xenopus          KVIKEKLGPPYGIKRLTYFCPVCQPQVKCDPSSSSL---  313
Monodelphis      QVMKDSFGPPGSFQRLTWWCPHCQPKAEEKVEVTQEQLP  337
Human            QVMKEAFGPEDGLQRLTWWCPQCQPQLSEEPEQCQFS--  332
Mouse            QVMKETFGPPDGLQRLTWWCPQCQPQLSSKGPQNLPSS-  329
Rat              QVMKETFGPPDGLQRLTWWCPQCQPQLSSEGPQNLLSS-  330
                 :*:*: :**  .::***::** ***: . .
```

Figure S4 Evolutionary conservation of predicted phosphosites in NEIL2 Alignment of *X. tropicalis*, *M. domestica*, *H. sapiens*, *M. musculus*, and *R. rattus* NEIL2 protein sequence. * fully conserved site : conservation between groups of strongly similar properties . conservation between groups of weakly similar properties. Black: predicted PKC phosphosites based on *in silico* predictions. Grey: predicted CDK5 phosphosites based on *in silico* predictions.

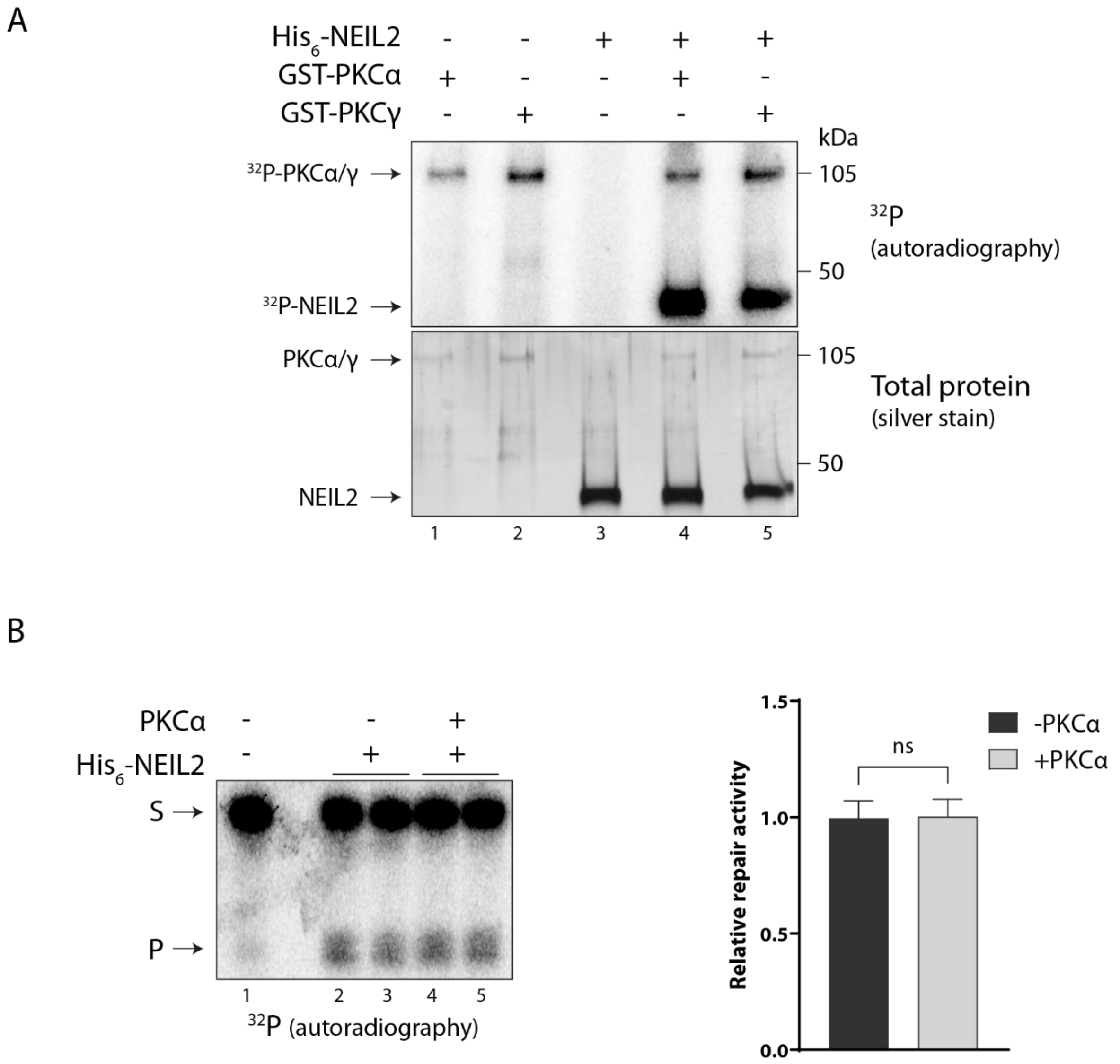

Figure S5 PKC alpha phosphorylates NEIL2 without altering NEIL2 repair activity *in vitro* A) 200 ng purified NEIL2 was incubated with 25 ng recombinant GST-tagged PKCγ or 25 ng recombinant GST-tagged PKCα as indicated in the presence of [^{32}P]-ATP for 45 min at 30°C and proteins were separated by SDS-PAGE for autoradiography. PKC undergoes autophosphorylation which can be seen as a band around 105 kDa. Phosphorylation of NEIL2 can be seen as a band close to 50 kDa. Total protein was stained by silver staining. B) Purified NEIL2 was incubated with recombinant PKCα in the presence of unlabelled ATP for 10 min at 30°C. Subsequently, non-phosphorylated (incubated for 10 min at 30°C without kinase) or phosphorylated NEIL2 was incubated with the 5'-end ^{32}P-labelled 5OHU B11 oligonucleotide substrate for 15 min. Substrate and repair product were separated in a denaturing polyacrylamide gel and visualized by autoradiography. S: substrate, P: product. Data is displayed as mean + SD and represent at least three independent experiments.

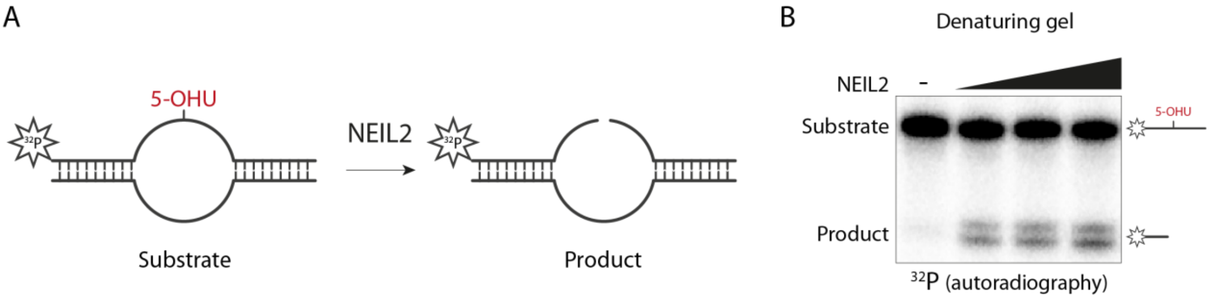

Figure S6 Principle of the NEIL2 DNA repair assay A) Purified recombinant NEIL2 is incubated with an oligonucleotide substrate containing a 5-hydroxyuracil (5-OHU) lesion in a 11 nt bubble. The substrate is 5'-end labelled with ^{32}P on the same strand as the lesion. NEIL2 repair activity towards the lesion results in excision of the damaged base generating an abasic site followed by incision of the DNA backbone resulting in a single-strand break. Hence, NEIL2 repair activity leads to a shorter 5'-end labelled strand as the repair product. B) The ^{32}P-labelled repair product can be separated from the ^{32}P-labelled substrate in a denaturing gel and visualized by autoradiography.

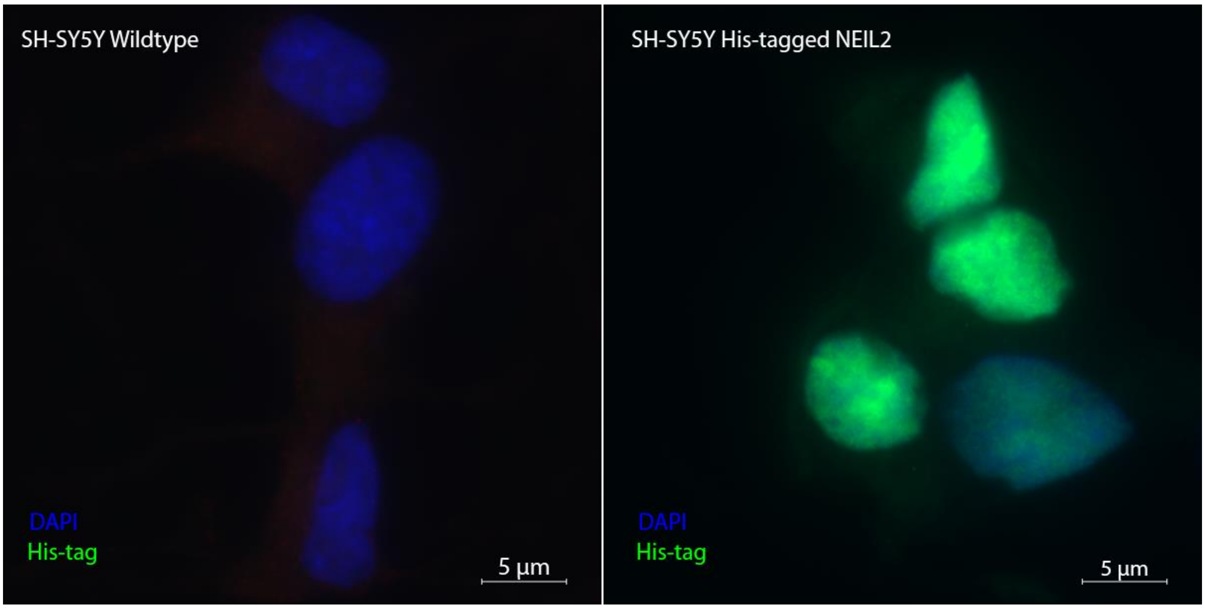

Figure S7 Specificity of His-tag antibody in immunofluorescence imaging SH-SY5Y cells, wildtype and His-tagged NEIL2 expressing, respectively, were fixed and stained with anti His-tag antibody (Cell Signaling, #12698).
