# Supplementary material for: Phosphorylation of the Human DNA Glycosylase NEIL2 Is Affected by Oxidative Stress and Modulates Its Activity
Source: Antioxidants (Basel). 2023 Feb 2;12(2):355. doi: 10.3390/antiox12020355 (PMC9952225; doi:10.3390/antiox12020355)
Supplement: Supplementary file 1 [file antioxidants-12-00355-s001.zip › Supplementary materials.pdf]

Supplementary materials

DNA purification and PCR

Genomic DNA was purified from $5x10^6$ cells with the QIAamp DNA mini kit (Qiagen) according to manufacturer's protocol. Genomic integration of the expression vector was confirmed by PCR with a forward primer targeting the cytomegalovirus (CMV) promoter (5'-CGCAAATGGGCGGTAGGCGTG-3') and a reverse primer targeting the bovine growth hormone polyadenylation (bGH-PolyA) signal (5'-TAGAAGGCACAGTCGAGG-3'), respectively. The CMV promoter and bGH-PolyA signal flank the ORF of the NEIL2 gene in the pcDNA3.1.D expression vector and are necessary for expression (Fig. S1A). Genomic integration of the vector results in a PCR product of 1394 bp with these primers (Fig. S1B). PCR was conducted with the Taq PCR kit (New England Biolabs), and reactions contained 250 ng genomic DNA, 1x reaction buffer, 200 µM dNTPs, 0.5 µM forward primer, 0.5 µM reverse primer, and 0.025U/µL Taq DNA polymerase. Genomic DNA from non-transfected SH-SY5Y cells was utilized as a negative control and purified pcDNA3.1.D expression vector as a positive control. The PCR cycling conditions were as follows: 1 cycle of 95°C for 30s, 30 cycles of 95°C for 15s, 65°C for 30s, and 68°C for 90s, and 1 cycle of 72°C for 5 min. PCR products were visualized on a 1% agarose gel.

Cell extracts for western blotting

Cell pellets were lysed in RIPA buffer (150 mM NaCl, 1% Triton X, 10 mM Tris-HCl pH 8.0, and 0.5% sodium deoxycholate, 1% protease inhibitor cocktail (#539134, Merck), 1% phosphatase inhibitor cocktail 2 (#P5726, Sigma), and 1% phosphatase inhibitor cocktail 3 (#P0044, Sigma)) followed by sonication and 30 min incubation on ice. Cell debris was removed by centrifugation at 16,000xg for 10 min at 4°C. Protein concentration was determined by Bradford protein assay (BioRad) and extracts were stored at -80°C until further analysis.

Bioinformatics

Amino acid sequence alignment was performed with Clustal Omega (v. 1.2.4) using NEIL2 protein sequence from human (Q969S2-1), mouse (Q6R2P8), rat (D4ABX3), frog (A9ULI4), and opossum (F7AMK3), respectively. Analysis of PKC isoform specificity on predicted NEIL2 phosphosites was conducted by use of experimentally determined protein kinase specificity available in KinaseNET (Kinexus Bioinformatics Corporation).
