# Supplementary material for: Phosphorylation of the Human DNA Glycosylase NEIL2 Is Affected by Oxidative Stress and Modulates Its Activity
Source: Antioxidants (Basel). 2023 Feb 2;12(2):355. doi: 10.3390/antiox12020355 (PMC9952225; doi:10.3390/antiox12020355)
Supplement: Supplementary file 1 [file antioxidants-12-00355-s001.zip › Table S1.pdf]

Name	Sequence
5-OHU B11	GCT TAG CTT GGA ATC GTA TCA TGT A**(5-OHU)**A CTC GTG TGC CGT GTA GAC CGT GCC
Control B11	GCT TAG CTT GGA ATC GTA TCA TGT A**C**A CTC GTG TGC CGT GTA GAC CGT GCC
B11 complementary	GGC ACG GTC TAC ACG GCA CAA ACA GCC CAC GGA TAC GAT TCC AAG CTA AGC

Table S1 List of oligonucleotides used for NEIL2 activity assay B11: 11 nt non-complementary sequence. The sequence is underlined and gives rise to an 11 nt single-stranded bubble structure after annealing. 5-OHU: 5-hydroxyuracil
